# Knemidocoptic Mange in Wild Golden Eagles, California, USA

**DOI:** 10.3201/eid2010.140504

**Published:** 2014-10

**Authors:** Aslı Mete, Nicole Stephenson, Krysta Rogers, Michelle G. Hawkins, Miranda Sadar, David Sanchez-Migallon Guzman, Douglas A. Bell, Kenneth S. Smallwood, Amy Wells, Jessica Shipman, Janet Foley

**Affiliations:** California Animal Health and Food Safety Laboratory, Davis, California, USA (A. Mete);; University of California, Davis (N. Stephenson, M.G. Hawkins, M. Sadar, D. Sanchez-Migallon Guzman, J. Foley);; California Department of Fish and Wildlife, Rancho Cordova, California, USA (K. Rogers);; East Bay Regional Park District, Oakland, California, USA (D.A. Bell);; Researcher and Ecologist, Davis (K.S. Smallwood);; Avian and Exotic Clinic of the Monterey Peninsula, Del Rey Oaks, California, USA (A. Wells);; SPCA for Monterey County, Salinas, California, USA (J. Shipman)

**Keywords:** ectoparasite, electron microscopy, golden eagle, *Knemidocoptes* sp., mange, raptor, skin disease, wild, parasites

## Abstract

During 2012–2013 in California, USA, 3 wild golden eagles were found with severe skin disease; 2 died. The cause was a rare mite, most closely related to *Knemidocoptes derooi* mites. Cautionary monitoring of eagle populations, habitats, and diseases is warranted.

Knemidocoptidae are mange mites that cause alopecia, acanthosis, hyperkeratosis, pruritus, feather damage, inappetence, and sometimes death in birds (*1*,*2*). Infestation is typically worse in older, injured, sick, stressed, or malnourished birds (*3*). Most reports describe *Knemidocoptes mutans* and *K. gallinae* mites as affecting gallinaceous birds and *K. pilae* and *K. jamaicensis* mites as affecting pet or exotic birds in captivity (*1*,*2*). Although rarely fatal, epizootics among wild birds have been described; most affected raptors have been in captivity or rehabilitation centers (*2*,*4*,*5*). We describe the pathology caused by a rare species of mange mite in 1 of 3 wild golden eagles (*Aquila chrysaetos*) during an outbreak of knemidocoptosis. 

## The Cases

During December 2012, eagle 1 was found in Hollister, California, after being struck by a car. The 3.5-kg bird was admitted to the SPCA for Monterey County, Salinas, California; it was prostrate from head trauma and died overnight. Examination revealed feather loss and scabbing on the head, neck, legs, and near the cloaca; microscopic examination of a skin scraping revealed mites.

During July 2013, eagle 2 was live-trapped in the Altamont Pass Wind Resource Area near Livermore, California. This subadult (*6*) female had skin lesions similar to those of eagle 1. After mites were detected, she received treatment at the Veterinary Medical Teaching Hospital, University of California, Davis, and underwent rehabilitation at the California Raptor Center. Skin scrapings with crusts were collected and stored at −20°C.

During August 2013, eagle 3 was found grounded in King City, California, and admitted to the SPCA for Monterey County. This 2.7-kg subadult male exhibited severe dehydration, weak and raspy respiration, and poor quality feathers. Mites and lesions similar to those on eagles 1 and 2 were found. Because of a poor prognosis, eagle 3 was euthanized and the carcass was stored at −20°C. 

**Figure 1:**
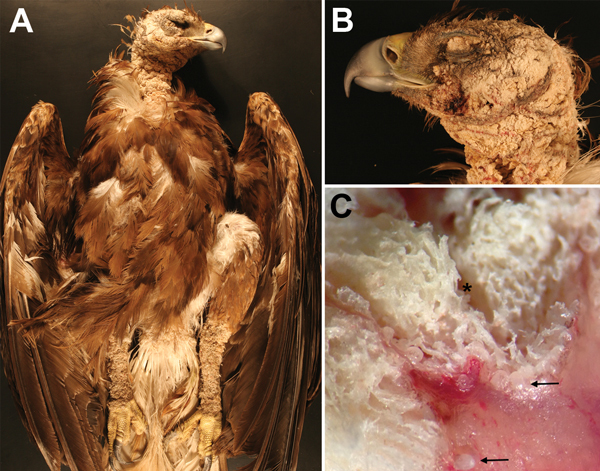
Golden eagle found grounded in King City, California, USA, during August 2013 (eagle 3). A) Photograph of diffuse crusting and thickening of the head, neck, and legs. B) Photograph showing severe obliteration of the skin over the eyelid and ear. C) Dissecting microscopic cross-section of the affected skin, showing thick trabeculae of keratin deposition (*) and white to transparent mites (arrows). Original magnification ×.

On August 15, 2013, a necropsy of eagle 3 was performed at the California Animal Health and Food Safety Laboratory System, Davis, California. The bird was thin and the skin of the head, neck, vent region, and legs (except feet) had rough, firm, thick, dry, scaly, gray-brown crusts (Figure 1, panel A). The skin thickness was up to 1 cm; the left eye and both ears were completely encrusted (Figure 1, panel B). Stereoscopy revealed white-to-transparent miliary bosselations beneath and within severely thickened keratin and a few crawling mites (Figure 1, panel C). The liver had disseminated pinpoint pale foci; lungs were heavy, wet, and dark red. Ancillary tests included aerobic bacterial culture of liver, spleen, and lung tissue and real-time PCR for *Salmonella* spp. in intestinal contents, for avian influenza virus and avian paramyxovirus-1 on an oropharyngeal swab sample, and for West Nile virus in kidney tissue. The femur was screened for chronic lead exposure, and liver tissue was screened for selenium, lead, manganese, cadmium, copper, iron, zinc, molybdenum, arsenic, mercury, and anticoagulant rodenticides. Bacteria and viruses were not detected, and all toxicants were either not detected or within acceptable range (except 2.1 ppm lead in bone and 0.01 ppm brodifacoum in liver).

Organ samples were fixed in formalin, embedded in paraffin, sectioned, and stained with hematoxylin and eosin. Histopathologic examination revealed generalized loss of feather follicles and numerous intracorneal mites. The epidermis was diffusely scalloped and covered by dense orthokeratotic lamellae, which formed serially aligned pouches within the stratum corneum; pouches contained developing and adult mites and eggs, mostly stacked and forming a honeycomb appearance. Subjacent epidermis was atrophied; acanthotic septae and papillary projections were overlain by orthokeratotic spires separating pouches. Coccoid bacteria and plant fragments were often associated with crusts. Throughout the dermis, small perivascular to interstitial heterophilic and pleocellular infiltrates were present. Acute necrotizing foci stippled with karyorrhexis and fibrin exudates and small plasmacytic infiltrates were found in myocardium, peripheral nerves and perineural tissues, liver, spleen, and lungs. Abundant protozoa were seen in lungs and heart; intense immunoreactivity to *Sarcocystis falcatula* protozoa was found in vessels, lungs, heart, and perineural tissues; occasional immunoreactivity was found in dermis and brain endothelium.

**Figure 2:**
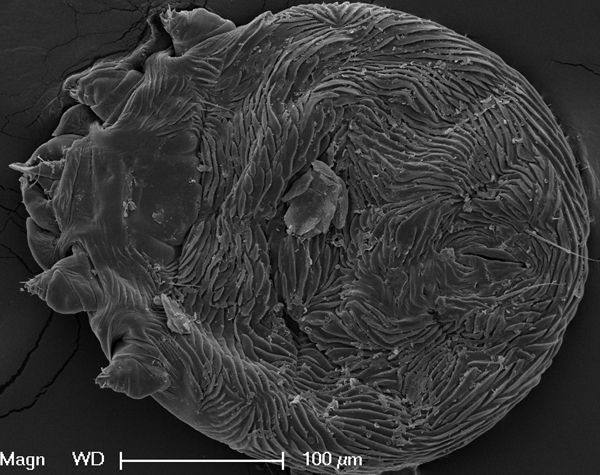
Electron microscopic image of mite, showing features consistent with *Knemidocoptes derooi* mites.

Frozen skin specimens from eagles 2 and 3 were submitted for parasite identification by electron microscopy and PCR. Mites were fixed with Karnovsky fixative in 0.1 mol/L Sorenson sodium phosphate buffer, washed with sodium phosphate, dehydrated in increasing ethanol to 100%, and dried in a Tousimis Research Corporation 931.GL Autosamdri Critical Point dryer (Rockville, MD, USA). The mites were mounted on aluminum stubs, sputter-coated with gold, and viewed on an FEI XL30 TMP scanning electron microscope (Eindhoven, the Netherlands). The mites closely resembled *K. derooi* mites on the basis of the dorsal striation pattern, relative size of the propodosoma, dorsoterminal location of the anus, and dorsal positioning of the anterior legs and ventral positioning of the posterior legs (Figure 2) (*7*).

DNA was extracted from 2 pools of 5–10 mites from eagles 2 and 3 by using a QIAGEN Tissue Kit (Valencia, CA, USA). Modified PCR amplification (*8*) of a fragment of the *cytochrome oxidase* subunit I gene included 12.5 μL of GoTaq Green Master Mix (Promega, Madison, WI, USA), 1.0 mol/L of each primer, 2.5 μL of water, and 5 μL of DNA. Bands of 715 bp were cleaned with a QIAGEN gel extraction kit before DNA sequencing (Davis Sequencing, Davis, CA, USA). A BLAST search was conducted to match the DNA sequences with those in GenBank. Sequence homology was 100% between the pools from both eagles and 88% to *K. jamaicensis* (JQ037816.1), the only *Knemidocoptes* mite in the database. The GenBank accession number for the golden eagle mites is KJ787640.

## Conclusions

The severity and diffuse distribution of skin lesions of these eagles suggest a possible serious, unique outbreak. Most knemidocoptic mites affect the face and cere, or legs and feet (called scaly face and scaly leg mites) (*1*,*2*). With such severe feather loss and crusting over the eyes and ears, irritation and limited thermoregulation probably decreased the ability of these eagles to feed and exacerbated clinical disease. The morphologic features of the mites were consistent with those of *K. derooi* mites, a species rarely reported and, to our knowledge, not reported in North America or on raptors (*7*). Although raptors sometimes have skin mites, such debilitating disease in otherwise healthy animals is highly atypical (*1*–*3*). Disease or exposure to contaminants might induce immunosuppression or toxicosis. Eagle 3 showed evidence of prior exposure to lead; although use of lead bullets has been banned in some areas of California since 2008, ingestion of spent lead ammunition in hunter-killed carcasses or viscera remains a threat to wildlife throughout much of the western United States (*9*). Brodifacoum, a second-generation anticoagulant rodenticide also identified in this eagle, has been found in golden eagles (*10*) and could induce sublethal effects and immunosuppression. The respiratory disease probably resulted from *S. falcatula* infection, which can produce fatal systemic disease in wild eagles (*11*).

The effects of environmental and climate change on bird–mite relationships might be a factor in the emergence of mange. Because *Knemidocoptes* spp. mites are transmitted by contact, stress induced by crowding, toxicosis, and concurrent pathogens might account for the severe disease among birds in close proximity, or these cases might reflect the emergence of a highly virulent mite (*2*,*3*). The density of golden eagles near the Altamont Pass Wind Resource Area is notably high (*12*), and this outbreak might reflect habitat changes bringing individuals into closer contact or increasing stress. Consistent multidisciplinary data collection is needed for identification of the mite’s host species range, ecology, and pathogenicity and for enhanced understanding of this possibly emerging fatal disease.
